# Corrigendum: Personalized digital intervention for depression based on social rhythm principles adds significantly to outpatient treatment

**DOI:** 10.3389/fdgth.2023.1136316

**Published:** 2023-03-16

**Authors:** Ellen Frank, Meredith L. Wallace, Mark J. Matthews, Jeremy Kendrick, Jeremy Leach, Tara Moore, Gabriel Aranovich, Tanzeem Choudhury, Nirav R. Shah, Zeenia Framroze, Greg Posey, Samuel A. Burgess, David J. Kupfer

**Affiliations:** ^1^ Department of Psychiatry, School of Medicine, University of Pittsburgh, Pittsburgh, United States; ^2^ HealthRhythms, Inc., Long Island, NY, United States; ^3^ School of Computer Science, University College Dublin, Dublin, County Dublin, Ireland; ^4^ Huntsman Mental Health Institute, University of Utah School of Medicine, Salt Lake, UT, United States; ^5^ Cornell Tech, New York, NY, United States; ^6^ School of Medicine, Stanford University, Stanford, CA, United States; ^7^ Sharecare, Atlanta, GA, United States; ^8^ School of Medicine, University of Pittsburgh, Pittsburgh, PA, United States

**Keywords:** treatment, digital intervention platform, passive monitoring, depressive symptoms, social rhythm disruption, social rhythm regularity, depression treatment

A Corrigendum on Personalized digital intervention for depression based on social rhythm principles adds significantly to outpatient treatment By Frank E, Wallace ML, Matthews MJ, Kendrick J, Leach J, Moore T, Aranovich G, Choudhury T, Shah NR, Framroze Z, Posey G, Burgess SA and Kupfer DJ. (2022) Front. Digit. Health 4:870522. doi: 10.3389/fdgth.2022.870522

**Incorrect Author Name**

In the published article, two author name initials were incorrectly written or omitted. “Mark L. Matthews” should be “Mark J. Matthews” and “Samuel Burgess” should be “Samuel A. Burgess”.

**Error in Figure/Table Legend**

In the published article, there was an error in the legend for Figure 8. The legend incorrectly stated that the figure displays “Means and standard errors of PHQ scores by study week–full sample”. The corrected legend appears below.

“Means and standard errors of PHQ scores by study week–depressed-at-entry sample”.

The authors apologize for these errors and state that they do not change the scientific conclusions of the article in any way. The original article has been updated.

